# Biomarkers predicting good prognosis among patients receiving immunosuppressive treatment in IgA nephropathy: the promising role of serum TGF-β1 and MCP-1

**DOI:** 10.3389/fimmu.2026.1852831

**Published:** 2026-06-24

**Authors:** Junseok Jeon, Youngmin Yoon, Kyungho Lee, Heasil Moon, Jung Eun Lee, Ghee Young Kwon, Wooseong Huh, Hye Ryoun Jang

**Affiliations:** 1 Division of Nephrology, Department of Medicine, Samsung Medical Center, Sungkyunkwan University School of Medicine, Seoul, Republic of Korea; 2 Division of Nephrology, Department of Medicine, Chosun University Hospital, Chosun University School of Medicine, Gwangju, Republic of Korea; 3 Department of Health Sciences and Technology, Samsung Advanced Institute for Health Sciences and Technology, Sungkyunkwan University, Seoul, Republic of Korea; 4 Department of Pathology, Samsung Medical Center, Samsung Biomedical Research Institute, Sungkyunkwan University School of Medicine, Seoul, Republic of Korea

**Keywords:** IgA nephropathy, immunosuppressive therapy, MCP-1, prognosis, TGF-β1

## Abstract

**Background:**

IgA nephropathy (IgAN) exhibits a highly variable clinical course. As responses to immunosuppressive therapy remain inconsistent, reliable prognostic markers are critically required to identify patients who can show a good response to immunosuppressive treatments.

**Methods:**

This prospective cohort study enrolled adults with biopsy-proven IgAN from 2010-2020. Good prognosis was defined as a ≥50% reduction in the urine protein-to-creatinine ratio (uPCR) or <0.5 g/g with preserved estimated glomerular filtration rate 1 year after biopsy. Serum and urine cytokines/chemokines (TGF-β1, MCP-1, RANTES, and VEGF) and intrarenal CD45^+^, CD3^+^, CD20^+^, and Ki-67^+^ cells were analyzed.

**Results:**

Among the 202 patients, 120 (59.4%) had a good prognosis and showed a significantly lower risk of end-stage kidney disease over a 10-year follow-up. Multivariable analyses identified immunosuppressive therapy, low histologic grade, lower uPCR, absence of hypertension, higher serum TGF-β1 levels, and reduced intrarenal CD45^+^ and CD3^+^ cell infiltration as independent predictors of good prognosis. Among patients receiving immunosuppressive therapy, higher serum TGF-β1 (adjusted odds ratio [aOR], 1.16; 95% confidence interval [CI], 1.04–1.31) and lower serum MCP-1 (aOR, 0.99; 95% CI, 0.97–1.00) independently predicted good prognosis, whereas less intrarenal CD20^+^ cell infiltration (aOR, 0.22; 95% CI, 0.07–0.73) independently predicted good prognosis in patients receiving supportive care only.

**Conclusions:**

Serum TGF-β1 and MCP-1 levels may serve as biomarkers of favorable prognosis among patients with IgAN receiving immunosuppressive therapy, whereas intrarenal CD20^+^ cell infiltration may reflect poor prognosis under supportive care. Integrating serum cytokine profiles and tissue immune signatures may help inform individualized treatment decisions for IgAN.

## 1 Introduction

IgA nephropathy (IgAN) is the most common form of primary glomerulonephritis worldwide (1, 2). The clinical course of IgAN is highly variable, ranging from asymptomatic cases to rapid progression to end-stage kidney disease (ESKD) (3, 4). Previous studies have reported that approximately 20% to 50% of patients with IgAN progress to ESKD within 20 to 30 years of diagnosis (5–7). This variability in disease outcomes, often leading to unfavorable renal prognosis, underscores the critical need to identify prognostic factors and optimize treatment strategies for patients with IgAN.

Supportive treatment for IgAN includes blood pressure control with renin-angiotensin system blockade, lifestyle modification such as low salt diet (8, 9), and potential sodium–glucose cotransporter-2 inhibitors (10, 11). Despite optimal supportive care, many patients remain at high risk of kidney failure, for which treatment with immunosuppressants has been recommended (8, 9, 12, 13). Systemic glucocorticoids have shown conflicting efficacy and are often limited by adverse effects, particularly an increased risk of infection (9, 13). Several novel agents are currently under clinical investigation, and the recent Kidney Disease: Improving Global Outcomes guideline recommends targeted-release oral budesonide; however, their clinical use is still limited by regulatory and cost barriers (14–16).

Given the potential adverse effects of immunosuppressive therapy, the identification of clinically relevant biomarkers is critically important to guide treatment decisions beyond traditional proteinuria-based criteria and to identify patients who are likely to achieve favorable outcomes with immunosuppressive therapy. The aim of this study was to identify biomarkers predictive of good prognosis in patients with IgAN, particularly among those receiving immunosuppressive therapy.

## 2 Methods

### 2.1 Study population

This prospective cohort study included patients aged 18–80 years who were diagnosed with biopsy-proven IgAN at Samsung Medical Center between 2010 and 2020. Patients who were lost to follow-up within 1 year after kidney biopsy and those who lacked a histologic grade were excluded. Patients were followed up for 1 to 10 years until the occurrence of the study endpoint or the date of the last follow-up, whichever occurred first. Patients were categorized according to treatment status within 1 year after kidney biopsy into the supportive care-only group, which included patients who were not treated with immunosuppressive agents, and the immunosuppressive therapy group, which included those treated with immunosuppressive agents such as corticosteroids, azathioprine, cyclosporine, mycophenolate mofetil (MMF), calcineurin inhibitor (CNIs; cyclosporine or tacrolimus), and cyclophosphamide, either alone or in combination. Among the 96 patients in this group, the proportion of treatment regimens was as follows: glucocorticoid monotherapy (n = 40), CNI monotherapy (n = 3), MMF monotherapy (n = 2), combination therapy with CNI and MMF (n = 4), combination therapy with CNI and azathioprine (n = 2), and combination therapy with glucocorticoids and other immunosuppressive agents (n = 45). Notably, the vast majority of patients (85 of 96, 88.5%) received glucocorticoids either as monotherapy or as part of combination regimens. This study was approved by the Institutional Review Board of Samsung Medical Center in compliance with the Declaration of Helsinki (No. 2010-03-007, 2015-06-140, and 2022-03-077). Informed consent was obtained from all patients.

### 2.2 Clinical parameters

Demographic and clinical data, such as age, sex, body mass index (BMI), blood pressure, medication use, and hypertension (HTN) status defined as previously documented diagnosis were extracted from electronic medical records. The mean arterial pressure (MAP) was calculated as the diastolic blood pressure + 1/3 × (systolic blood pressure − diastolic blood pressure). Laboratory parameters, including hemoglobin, blood urea nitrogen, serum creatinine, microscopic examination of urine for red blood cell (RBCs), and spot urine protein-to-creatinine ratio (uPCR), were obtained at the time of kidney biopsy. Anemia was defined as hemoglobin level <13.5 mg/dL in men and <12 mg/dL in women. Negative or minimal hematuria was defined as 0–11 RBCs per high-power field. The estimated glomerular filtration rate (eGFR) was calculated using the 2009 Chronic Kidney Disease Epidemiology Collaboration equation (17).

### 2.3 Histologic grade

Histologic grade was assessed using the Haas classification by an independent pathologist who was blinded to the study group (18). We categorized grades I, II, and III as low and grades IV and V as high.

### 2.4 Serum and urine cytokines/chemokines

Serum and urine samples collected on the day of kidney biopsy were analyzed for 193 of the 202 patients; samples from the remaining 9 patients were excluded due to insufficient sample volume or inadequate deep-freezer storage. Cytokine and chemokine analyses were performed using cases meeting analysis eligibility. The levels of cytokines and chemokines, including regulated on activation, normal T cell expressed and secreted (RANTES), monocyte chemoattractant protein-1 (MCP-1), transforming growth factor-β1 (TGF-β1), and vascular endothelial growth factor (VEGF), were measured using the R&D DuoSet ELISA kit (R&D Systems, Minneapolis, MN, USA).

### 2.5 Analyses of intrarenal leukocytes and proliferating cells

Formalin-fixed kidney tissues were subjected to immunohistochemical staining for CD45, CD3, CD20, and Ki-67 (Dako, Glostrup, Denmark). The samples were deparaffinized using xylene and rehydrated using a graded series of ethanol. Antigen retrieval was performed using Zytomed Systems HIER citrate buffer (Zytomed Systems, Berlin, Germany). The samples were then subjected to endogenous peroxidase blocking using REAL™ Peroxidase-Block solution (Dako) at room temperature. The slides were incubated overnight at 4°C with a protein block serum-free ready-to-use solution (Dako). On the following day, the slides were incubated with primary antibodies for 1h at room temperature, washed with phosphate-buffered saline, and then incubated with secondary antibodies for 30min at room temperature using the REAL™ EnVision™ detection system (Dako). Color development was performed using 3,3′-diaminobenzidine tetrahydrochloride (Dako) for CD45, CD3, CD20 and Ki-67. The slides were counterstained with Mayer’s hematoxylin (Dako). The quantification of CD45^+^, CD3^+^, CD20^+^, and Ki-67^+^ cells was performed using QuPath software version 0.5.1 (University of Edinburgh, Edinburgh, UK), and the results were expressed as the percentage of positive cells among the total nucleated cells.

### 2.6 Outcomes

The primary outcome was good prognosis. Patients were divided into two prognostic groups based on the changes in proteinuria and eGFR for 1 year after the kidney biopsy: control and good-prognosis groups. Good prognosis was defined as either a reduction in uPCR≥50% or uPCR<0.5 g/g, along with an eGFR≥60 mL/min/1.73 m^2^ or a reduction of <5 mL/min/1.73 m^2^ from baseline. The control group comprised patients who did not meet the criteria for good prognosis. The secondary outcomes were ESKD and a composite renal outcome of ESKD or eGFR decline>50% of the baseline. ESKD was defined as eGFR ≤ 5 mL/min/1.73m^2^ or the need for renal replacement therapy, including hemodialysis, peritoneal dialysis, or kidney transplantation.

### 2.7 Statistical analyses

Continuous variables are reported as mean ± standard deviation, and categorical variables are reported as number (percentage). Group comparisons were performed using Student’s *t*-test or the Mann-Whitney U test for continuous variables and Pearson’s chi-square test or Fisher’s exact test for categorical variables. Longitudinal changes in eGFR were evaluated using a linear mixed-effects model with random intercepts for patients, including fixed effects for time, group, and time × group interactions. *Post-hoc* pairwise comparisons at each time point were performed using the Sidak correction. For patients who reached ESKD, the eGFR was assumed to be 5 mL/min/1.73m^2^. The time to ESKD or composite renal outcome was illustrated using the Kaplan-Meier method, and intergroup differences were assessed using the log-rank test. Multivariable analyses were performed using variables with *P* < 0.1 in the univariable analyses. To assess the robustness of the multivariable logistic regression models, we evaluated multicollinearity using variance inflation factors and confirmed that all values were below 2.0 (**Supplementary Table 1**). To address potential overfitting given the limited events per variable in the subgroup analyses, we examined the stability of the results using parsimonious models with reduced covariates. The predictive performance was evaluated using the area under the receiver operating characteristic curve (AUROC) derived from multivariable logistic regression models. Internal validation was performed using bootstrap resampling with 1,000 iterations to estimate the optimism-corrected AUROC. Differences in AUROCs between nested models were compared using the bootstrap method with 1,000 resamples. The incremental predictive value of biomarkers was further assessed using the continuous net reclassification improvement (NRI) and integrated discrimination improvement (IDI), with 95% confidence intervals derived from 1,000 bootstrap resamples. Internal validation was performed using bootstrap resampling with 1,000 iterations to estimate the optimism-corrected AUROC. All statistical analyses were performed using IBM SPSS version 27.0.0.0 (IBM Corp., Armonk, NY, USA), GraphPad Prism (GraphPad Software, San Diego, CA, USA), and R version 4.4.3 (R Foundation for Statistical Computing, Vienna, Austria). A two-sided *P* < 0.05 was considered statistically significant.

## 3 Results

### 3.1 Characteristics and treatment of study participants

A total of 259 patients were initially included, of whom 38 were lost to follow-up and 19 lacked histologic grade; finally, 202 patients were included in the analysis (100 in the control group and 102 in the good-prognosis group). Baseline characteristics are summarized in **Table 1**. The mean age was 43 ± 13.1 years, and 100 (49.5%) were male. No significant differences were noted between the groups in mean age, proportion of male patients, or BMI. The good-prognosis group showed a lower prevalence of HTN (good-prognosis vs. control: 33.3% vs. 58.0%; *P* < 0.001), lower MAP (good-prognosis vs. control: 89 ± 10.8 vs. 94 ± 10.9 mmHg; *P* = 0.006), higher eGFR (good-prognosis vs. control: 68.6 ± 30.71 vs. 56.9 ± 29.37 mL/min/1.73 m^2^; *P* < 0.01), and lower uPCR (good-prognosis vs. control: 1.6 ± 1.56 vs. 2.1 ± 1.56 g/g; *P* = 0.04) than those in the control group. The proportion of patients with anemia and negative or minimal hematuria was comparable between the groups. High histologic grade (Haas grade IV or V) was more common in the control group than in the good-prognosis group (control vs. good-prognosis: 59.0% vs. 40.2%; *P* = 0.008). Additionally, the use of immunosuppressive therapy was significantly higher in the good-prognosis group than in the control group (good-prognosis vs. control: 56.9% vs. 38.0%; *P* = 0.007).

**Table 1:** Baseline characteristics of study participants.

Study participants (n = 202)	Control (n = 100)	Good prognosis (n = 102)	P-value
Age, years	44.3 ± 12.7	42.0 ± 13.3	0.208
Male sex (%)	48 (48)	52 (51.0)	0.672
BMI kg/m^2^	23.4 ± 3.5	23.4 ± 4.0	0.937
HTN	58 (58.0)	34 (33.3)	<0.001
MAP, mmHg	94.5 ± 10.9	89.9 ± 10.8	0.003
Anemia^a^(%)	59 (59.0)	50 (49.0)	0.155
eGFR mL/min/1.73m^2^	56.9 ± 29.4	68.6 ± 30.7	0.006
uPCR, g/g	2.1 ± 1.6	1.6 ± 1.6	0.041
Negative or minimal hematuria^b^(%)	31 (31.0)	22 (21.6)	0.128
High histologic grade^c^(%)	59 (59.0)	41 (40.2)	0.008
Immunosuppressive therapy (%)	38 (38.0)	58 (56.9)	0.007

Values for continuous variables are reported as mean ± standard deviation and categorical variables as number (percentage).

^a^ Anemia was defined as hemoglobin level <13.5 mg/dL in men and <12 mg/dL in women.

^b^ 0–11 red blood cells per high-power field.

^c^ Haas grade IV or V.

BMI, body mass index; HTN, hypertension; MAP, mean arterial pressure; eGFR, estimated glomerular filtration rate; uPCR, urine protein-to-creatinine ratio.

The characteristics of patients who received immunosuppressive therapy and those who received supportive care only are presented in **Supplementary Tables 2**, **3**, respectively. In the immunosuppressive therapy and supportive care-only cohorts, the baseline differences between the two prognostic groups were consistent with those observed in the overall population.

### 3.2 Good prognosis criteria as a surrogate marker of long-term prognosis

**Figure 1:**
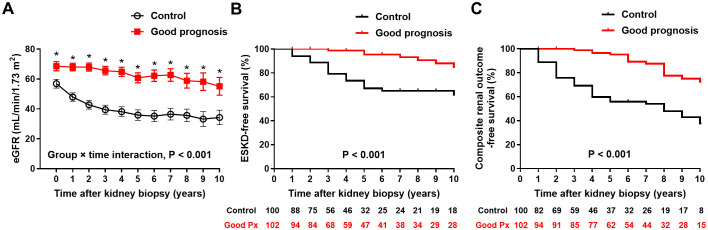
Comparison of renal outcomes between the good-prognosis and control groups. **(A)** Longitudinal change in eGFR over the follow-up period in the good-prognosis (red line) and control (black line) groups. Data are reported as mean ± standard error of the mean. Group differences in longitudinal eGFR changes were assessed using a mixed-effects model with Sidak-adjusted multiple comparisons. **(B)** Kaplan-Meier survival curves for the cumulative incidence of ESKD. **(C)** Kaplan-Meier survival curves for the composite renal outcome, defined as ESKD or a ≥50% decline in eGFR from baseline. Differences between groups in the Kaplan-Meier survival curves were assessed using the log-rank test. *P < 0.05. eGFR, estimated glomerular filtration rate; ESKD; end-stage kidney disease; Px, prognosis.

Good prognosis at 1 year after kidney biopsy was a strong predictor of a long-term good prognosis. The control group showed a rapid decline in eGFR during the first 5 years, whereas the good-prognosis group exhibited a more gradual decline throughout the entire follow-up period, resulting in a consistently significant difference in eGFR between the two groups (**Figure 1A**). ESKD-free survival and composite renal outcome-free survival were significantly different between the good-prognosis and control groups (**Figures 1B, C**).

### 3.3 Clinical and histologic factors associated with good prognosis

Among all patients, lower MAP, absence of HTN, higher eGFR, lower uPCR, low histologic grade, and immunosuppressive therapy were significantly associated with a good prognosis in the univariable analysis. The absence of HTN (adjusted odds ratio [aOR], 0.48; 95% confidence interval [CI], 0.24–0.96; *P* = 0.04), lower uPCR (aOR, 0.78; 95% CI, 0.62–0.98; *P* = 0.03), low histologic grade (aOR, 0.39; 95% CI, 0.20–0.79; *P* = 0.008), and use of immunosuppressive therapy (aOR, 4.55; 95% CI, 2.20–9.73; *P* < 0.001) were independently associated with a good prognosis in the multivariable analysis (**Table 2**). Among patients receiving immunosuppressive therapy, low histologic grade (aOR, 0.30; 95% CI, 0.11–0.87; *P* = 0.02) was the only independent predictor of good prognosis in the multivariable analysis (**Table 2**). In contrast, among patients receiving supportive care only, lower uPCR (aOR, 0.53; 95% CI, 0.31–0.92; *P* = 0.02) was the only independent predictor of good prognosis in the multivariable analysis (**Table 2**). When Haas grades IV and V were analyzed separately, patients with grade IV showed a higher good prognosis rate than those with grade V in both treatment groups (immunosuppressive therapy: 58.3% vs 40.0%; supportive care: 33.3% vs 13.3%). The ESKD rate was substantially higher in Haas grade V than in grade IV among patients receiving immunosuppressive therapy (52.0% vs 19.4%) (**Supplementary Table 4**).

**Table 2:** Predictors of good prognosis at 1 year after kidney biopsy.

Variables	Univariable		Multivariable	
	OR (95% CI)	p value	OR (95% CI)	p value
All patients				
Age, years	0.99 (0.97–1.01)	0.208		
Male sex	1.13 (0.65–2.00)	0.672		
BMI, kg/m^2^	1.00 (0.93-1.08)	0.937		
MAP, mmHg	0.96 (0.94–0.99)	0.004	0.99 (0.96–1.03)	0.644
HTN	0.36 (0.20–0.64)	< 0.001	0.48 (0.24–0.96)	0.039
Anemia	0.67 (0.38–1.17)	0.155		
eGFR, mL/min/1.73 m^2^	1.01 (1.00–1.02)	0.008	1.01 (1.00–1.02)	0.123
uPCR, g/g	0.83 (0.69–1.00)	0.045	0.78 (0.62–0.98)	0.030
Negative or minimal hematuria	1.63 (0.87–3.08)	0.129		
High histologic grade	0.47 (0.27–0.82)	0.008	0.39 (0.20–0.79)	0.008
Immunosuppressive therapy	2.15 (1.23–3.78)	0.008	4.55 (2.20–9.43)	<0.001
Patients receiving immunosuppressive therapy				
Age, years	1.00 (0.97–1.04)	0.942		
Male sex	1.11 (0.49–2.52)	0.807		
BMI, kg/m^2^	1.06 (0.94–1.20)	0.348		
MAP, mmHg	0.96 (0.93–1.00)	0.037	0.98 (0.94–1.03)	0.421
HTN	0.35 (0.15–0.83)	0.016	0.42 (0.16–1.14)	0.087
Anemia	0.44 (0.18–1.07)	0.070	0.47 (0.16–1.39)	0.172
eGFR, mL/min/1.73 m^2^	1.02 (1.00–1.04)	0.024	1.01 (0.99–1.03)	0.458
uPCR, g/g	0.82 (0.65–1.03)	0.081	0.87 (0.66–1.15)	0.338
Negative or minimal hematuria	1.39 (0.47–4.09)	0.549		
High histologic grade	0.31 (0.12–0.78)	0.013	0.30 (0.11–0.85)	0.023
Patients receiving supportive care only				
Age, years	0.97 (0.95–1.00)	0.077	0.99 (0.96–1.03)	0.686
Male sex	1.01 (0.47–2.20)	0.976		
BMI, kg/m^2^	0.99 (0.90–1.09)	0.805		
MAP, mmHg	0.94 (0.90–0.99)	0.009	1.00 (0.94–1.07)	0.977
HTN	0.37 (0.17–0.83)	0.016	0.55 (0.18–1.65)	0.285
Anemia	0.69 (0.32–1.51)	0.356		
eGFR, mL/min/1.73 m^2^	1.02 (1.00–1.03)	0.016	1.00 (0.98–1.02)	0.890
uPCR, g/g	0.48 (0.29–0.80)	0.004	0.53 (0.31–0.92)	0.024
Negative or minimal hematuria	0.44 (0.18–1.04)	0.061	0.45 (0.16–1.25)	0.125
High histologic grade	0.34 (0.14–0.79)	0.013	0.46 (0.17–1.26)	0.129

BMI, body mass index; eGFR, estimated glomerular filtration rate; HTN, hypertension; MAP, mean arterial pressure; uPCR, urine protein-to-creatinine ratio.

### 3.4 Serum and urine cytokines/chemokines for predicting good prognosis

Among all patients, serum TGF-β1 levels were significantly higher in the good-prognosis group than in the control group, whereas urine TGF-β1 were higher in the control group (**Supplementary Figure 1**). In the univariable analysis, higher serum TGF-β1 levels and lower urine TGF-β1 levels were associated with a good prognosis (**Table 3**). However, after adjusting for clinical variables and histologic grade, only higher serum TGF-β1 was associated with good prognosis (aOR, 1.07; 95% CI, 1.01–1.12; *P* = 0.02). Serum and urine levels of RANTES and VEGF were comparable between the groups.

**Table 3:** Serum and urine cytokines/chemokines for predicting good prognosis.

Variables	Unadjusted		Adjusted	
	OR (95% CI)	p value	OR (95% CI)	p value
All patients				
TGF-β1 (serum), pg/mL	1.05 (1.01 – 1.09)	0.014	1.07 (1.01 – 1.12)	0.016
TGF-β1/Cr (urine), pg/mg	0.99 (0.97 – 1.00)	0.028	0.99 (0.97 – 1.01)	0.302
MCP-1 (serum), pg/mL	1.00 (0.99 – 1.00)	0.176	1.00 (0.99 – 1.00)	0.235
MCP-1/Cr (urine), pg/mg	0.98 (0.93 – 1.03)	0.460	0.97 (0.90 – 1.04)	0.361
RANTES (serum), pg/mL	1.00 (1.00 – 1.00)	0.649	1.00 (1.00 – 1.00)	0.414
RANTES/Cr (urine), pg/mg	1.65 (0.24 – 11.26)	0.611	1.34 (0.11 – 16.25)	0.818
VEGF (serum), pg/mL	1.00 (1.00 – 1.01)	0.189	1.00 (1.00 – 1.01)	0.591
VEGF/Cr (urine), pg/mg	1.42 (0.76 – 2.65)	0.267	1.36 (0.63 – 2.92)	0.430
Patients receiving immunosuppressive therapy				
TGF-β1 (serum), pg/mL	1.10 (1.03 – 1.19)	0.007	1.16 (1.04 – 1.31)	0.010
TGF-β1/Cr (urine), pg/mg	0.98 (0.95 – 1.00)	0.027	0.99 (0.96 – 1.02)	0.473
MCP-1 (serum), pg/mL	0.99 (0.98 – 1.00)	0.017	0.99 (0.97 – 1.00)	0.016
MCP-1/Cr (urine), pg/mg	0.94 (0.88 – 1.01)	0.070	0.96 (0.89 – 1.05)	0.362
RANTES (serum), pg/mL	1.00 (1.00 – 1.00)	0.447	1.00 (1.00 – 1.00)	0.172
RANTES/Cr (urine), pg/mg	0.34 (0.03 – 3.52)	0.368	0.33 (0.10 – 12.28)	0.549
VEGF (serum), pg/mL	1.00 (1.00 – 1.01)	0.311	1.00 (0.99 – 1.01)	0.757
VEGF/Cr (urine), pg/mg	0.95 (0.42 – 2.14)	0.891	1.33 (0.43 – 4.11)	0.622
Patients receiving supportive care only				
TGF-β1 (serum), pg/mL	1.04 (0.99 – 1.09)	0.167	1.03 (0.95 – 1.10)	0.504
TGF-β1/Cr (urine), pg/mg	0.99 (0.97 – 1.01)	0.247	1.00 (0.98 – 1.02)	0.998
MCP-1 (serum), pg/mL	1.00 (0.99 – 1.01)	0.779	1.00 (0.99 – 1.01)	0.648
MCP-1/Cr (urine), pg/mg	0.96 (0.82 – 1.11)	0.545	1.08 (0.88 – 1.33)	0.455
RANTES (serum), pg/mL	1.00 (1.00 – 1.00)	0.973	1.00 (1.00 – 1.00)	0.895
RANTES/Cr (urine), pg/mg	18.0 (0.44 – 736.75)	0.127	5.16 (0.05 – 550.49)	0.491
VEGF (serum), pg/mL	1.01 (1.00 – 1.01)	0.180	1.00 (1.00 – 1.01)	0.389
VEGF/Cr (urine), pg/mg	1.88 (0.67 – 5.26)	0.232	1.42 (0.42 – 4.77)	0.568

Adjusted for age, sex, BMI, MAP, hypertension, anemia, eGFR, uPCR, hematuria, and histologic grade (plus immunosuppressive therapy in the overall cohort).

**Figure 2:**
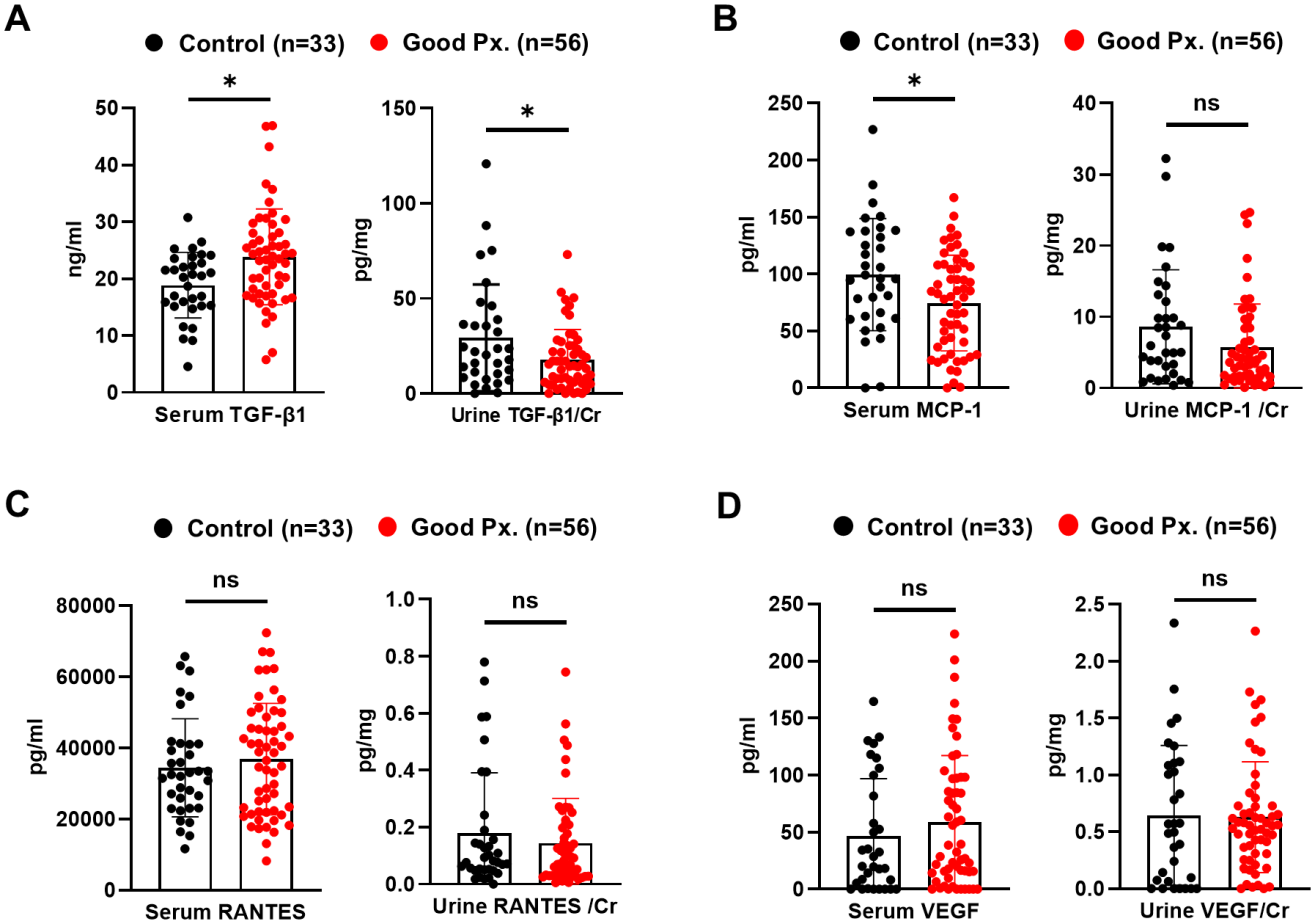
Serum and urine cytokine/chemokine levels in patients receiving immunosuppressive therapy. Comparison of serum and urine cytokine/chemokine levels between the good-prognosis (red) and control (black) groups among patients treated with immunosuppressive therapy. Panels show concentrations of **(A)** TGF-β1, **(B)** MCP-1, **(C)** RANTES, and **(D)** VEGF. Urine cytokine/chemokine levels were normalized to the urine Cr concentration. Data are reported as individual patient values overlaid on bar plots representing the mean ± standard error of the mean. Statistical comparisons were performed using Student’s *t*-test. For all graphs, NS, not significant; *P < 0.05. TGF-β1, transforming growth factor-β1; Cr, creatinine; MCP-1, monocyte chemoattractant protein-1; RANTES, regulated on activation, normal T cell expressed and secreted; VEGF, vascular endothelial growth factor; Px, prognosis.

Among patients who received immunosuppressive therapy, the good-prognosis group showed significantly higher serum TGF-β1, and lower urine TGF-β1 and serum MCP-1 levels (**Figure 2**). In the multivariable analysis, higher serum TGF-β1 levels (aOR, 1.16; 95% CI, 1.04–1.31; *P* = 0.01) and lower serum MCP-1 levels (aOR, 0.99; 95% CI, 0.97–1.00; *P* = 0.02) were independently associated with good prognosis after adjusting for clinical variables and histologic grade (**Table 3**). Serum TGF-β1 remained a significant predictor of good prognosis in parsimonious models adjusted for eGFR, uPCR, and histologic grade only (OR, 1.11; 95% CI, 1.03–1.20; P = 0.009), and serum MCP-1 also retained significance in the same parsimonious model (OR, 0.99; 95% CI, 0.98–1.00; P = 0.045; **Supplementary Table 5**). Kaplan-Meier analysis showed that those in the high serum TGF-β1 group had a trend toward a lower risk of ESKD (*P* = 0.19 using the log-rank test; *P* = 0.03 using the Gehan-Breslow-Wilcoxon test) and a significantly lower risk of the composite renal outcome (*P* = 0.02 using the log-rank test; **Supplementary Figure 2A**). The significant Gehan-Breslow-Wilcoxon result indicated that early outcome differences were more pronounced. In contrast, higher serum MCP-1 levels tended to be associated with a higher risk of ESKD (*P* = 0.08) but were not associated with the composite renal outcome (*P* = 0.13) (**Supplementary Figure 2B**).

**Figure 3:**
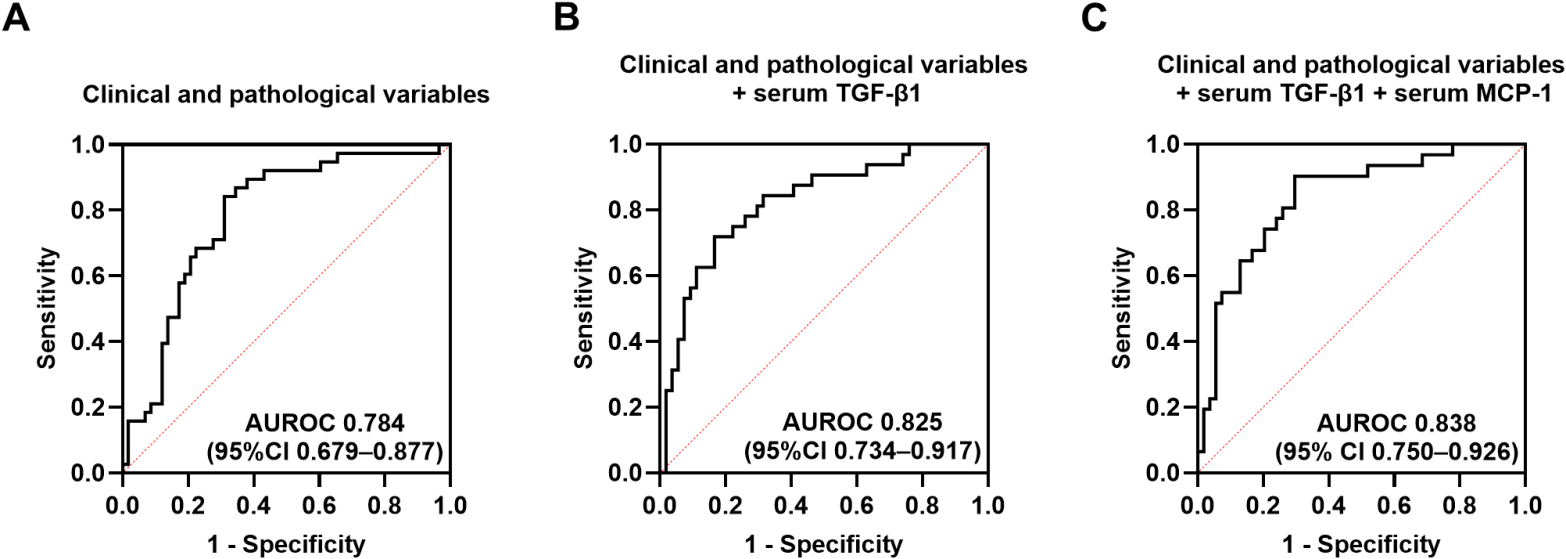
Predicting good prognosis in patients receiving immunosuppressive therapy. ROC curves comparing the predictive performance of logistic regression models for good prognosis among patients treated with immunosuppressive therapy. **(A)** ROC curve for the baseline model, which includes only established clinical variables and pathologic grade. **(B)** ROC curve for the model incorporating serum TGF-β1 in addition to the baseline model. **(C)** ROC curve for the model incorporating serum TGF-β1 and serum MCP-1, in addition to the baseline model. The dashed diagonal line represents the line of no discrimination (AUROC = 0.5). ROC, receiver operating characteristic curve; AUROC, area under the receiver operating characteristic curve; TGF-β1, transforming growth factor-β1; MCP-1, monocyte chemoattractant protein-1.

We constructed logistic regression-based predictive models to determine the utility of serum TGF-β1 and MCP-1 levels in predicting good prognosis among patients receiving immunosuppressive therapy. For predicting good prognosis, the model comprising only clinical variables and pathologic grade demonstrated an AUROC of 0.784 (95% CI, 0.679–0.877) (**Figure 3A**). When serum TGF-β1 alone and serum TGF-β1 and serum MCP-1 were added to the model, the AUROC increased to 0.825 (95% CI, 0.734–0.917) and 0.8381 (95% CI, 0.750–0.926), respectively (**Figures 3B, C**). Bootstrap internal validation yielded an optimism-corrected AUROC of 0.751 for the combined model including serum TGF-β1 and MCP-1, compared with 0.737 for the clinical and pathologic model alone (**Supplementary Table 6**). Among patients receiving supportive care only, serum and urine cytokines/chemokines were not associated with good prognosis, regardless of the adjustment for clinical variables and histologic grade (**Table 3**). The improvement in AUROC from the baseline model was 0.042 (P = 0.180) with the addition of serum TGF-β1 alone and 0.061 (P = 0.062) with the addition of both serum TGF-β1 and MCP-1 (**Supplementary Table 7**). The measures of reclassification improvement demonstrated significant added predictive value: the continuous net reclassification improvement was 0.661 (95% CI, 0.229–1.070; P = 0.003) and the integrated discrimination improvement was 0.123 (95% CI, 0.056–0.199; P < 0.001) when comparing the combined model with the clinical and pathologic model alone (**Supplementary Table 8**).

When the biomarker associations were examined separately by Haas grade among patients receiving immunosuppressive therapy, serum TGF-β1 levels were consistently higher in the good prognosis group than in the control group in both Haas grade IV (23.4 ± 6.8 vs 21.0 ± 3.6 ng/mL) and grade V (26.4 ± 9.2 vs 17.9 ± 6.9 ng/mL). In contrast, the pattern for serum MCP-1 was less consistent across grades (**Supplementary Table 4**).

### 3.5 Intrarenal leukocytes infiltration and Ki-67-positive cells for predicting good prognosis

**Figure 4:**
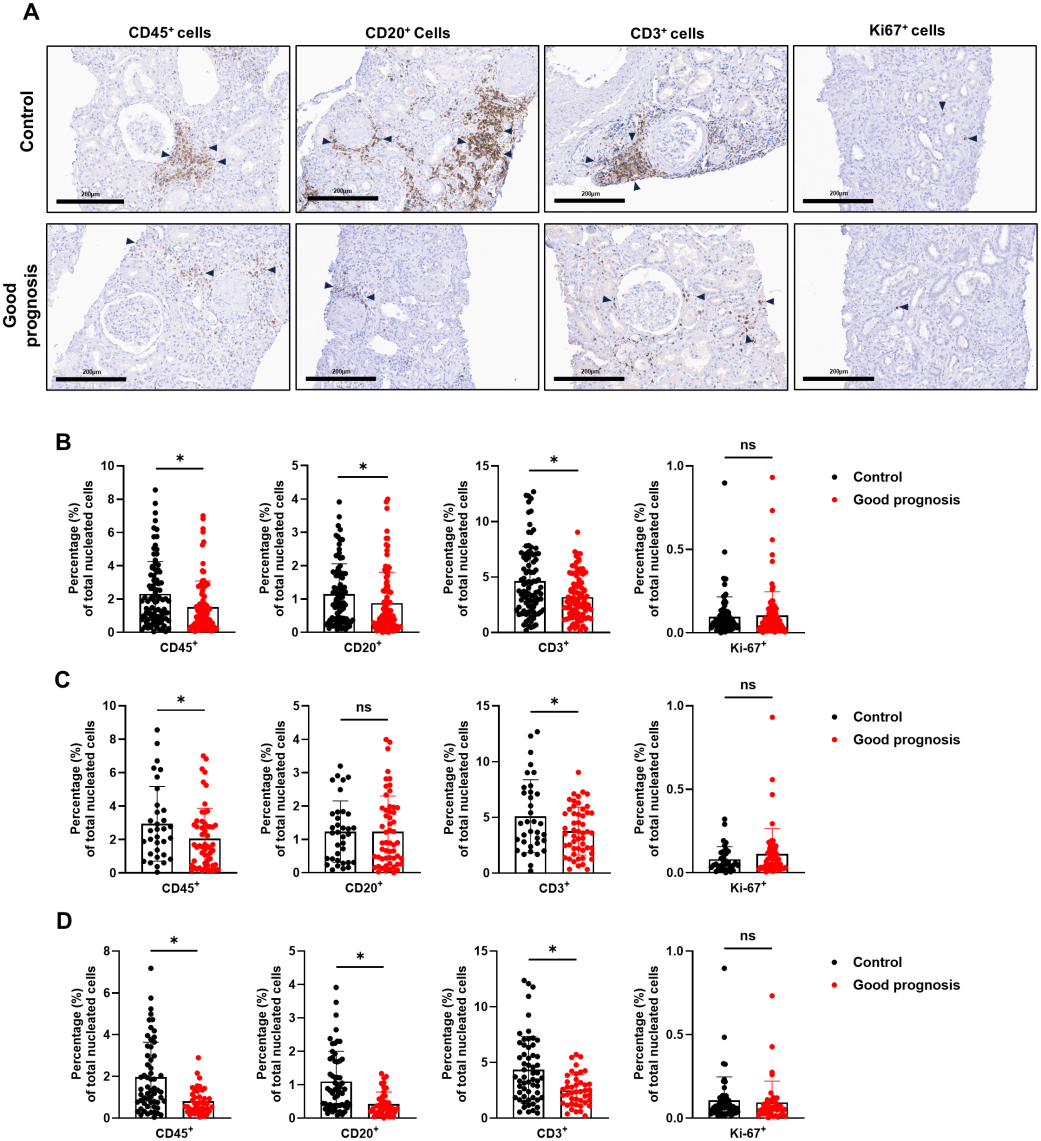
Intrarenal immune cell infiltration and proliferating tubular cells stratified by prognosis group and treatment strategy. **(A)** Representative immunohistochemistry images of CD45^+^, CD20^+^, CD3^+^, and Ki-67^+^ cells in kidney biopsy specimens from the control and good-prognosis groups. Arrowheads indicate positively stained cells. Scale bars = 200 μm. **(B–D)** Quantitative comparison of the proportion of positive cells among total nucleated cells between the control (black) and good-prognosis (red) groups: **(B)** all patients, **(C)** patients receiving immunosuppressive therapy, and **(D)** patients receiving supportive care only. Data are reported as individual patient values overlaid on bar plots representing the mean ± standard error of the mean. Statistical comparisons were performed using Student’s *t*-test. For all graphs, NS, not significant; *P < 0.05.

**Figure 5:**
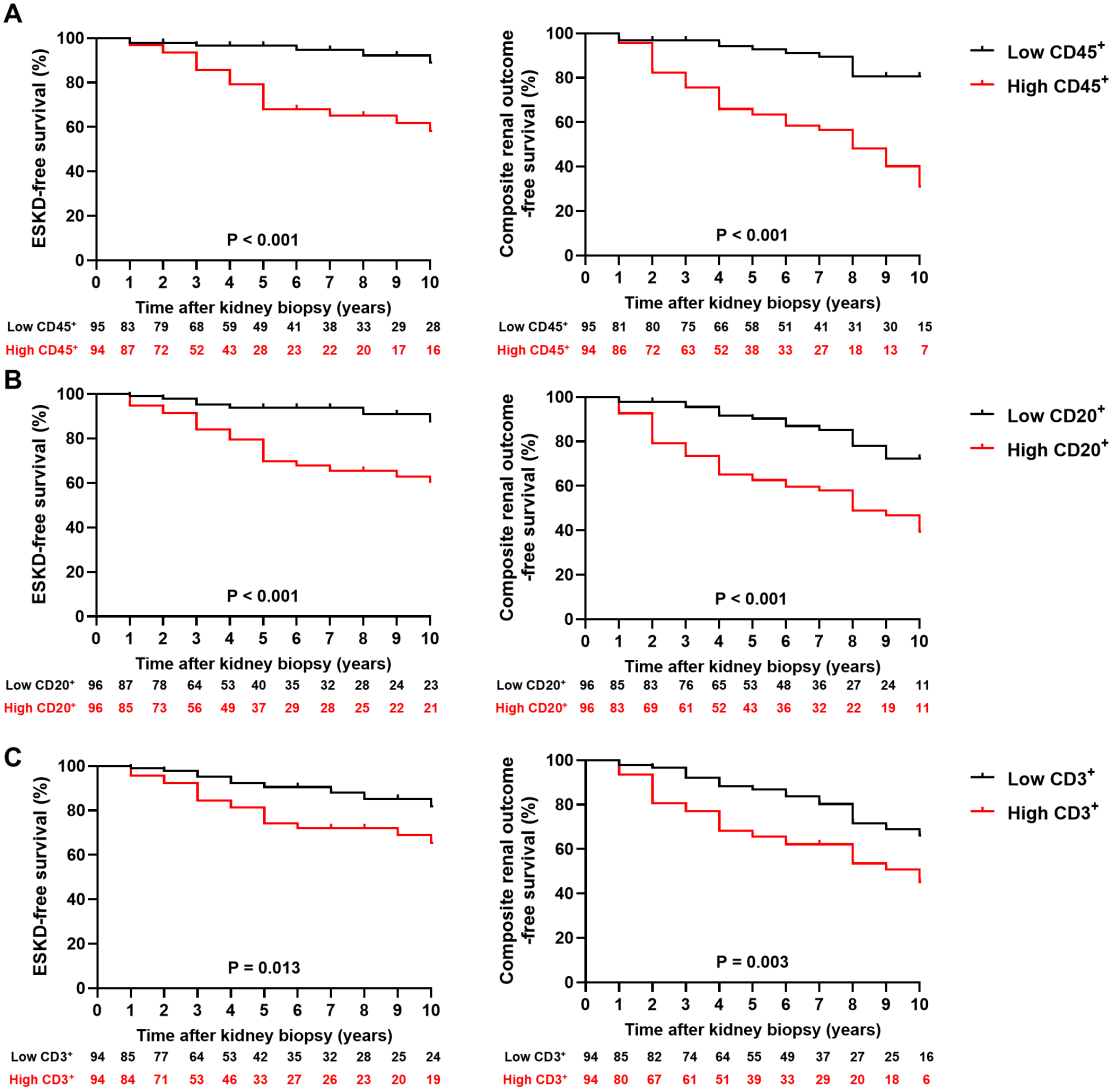
Kidney outcomes according to intrarenal immune cell infiltration. Kaplan-Meier survival curves for kidney outcomes stratified by median levels of intrarenal immune cell infiltration. **(A)** ESKD-free survival (left) and composite renal outcome-free survival (right) according to CD45^+^ proportion (low, n = 95; high, n = 94). **(B)** ESKD-free survival (left) and composite renal outcome-free survival (right) according to CD20^+^ proportion (low, n = 96; high, n = 96). **(C)** ESKD-free survival (left) and composite renal outcome-free survival (right) according to CD3^+^ proportion (low, n = 94; high, n = 94). *P-*values were calculated using the log-rank test. ESKD, end-stage kidney disease; TGF-β1, transforming growth factor-β1; MCP-1, monocyte chemoattractant protein-1.

CD45^+^, CD20^+^, and CD3^+^ cell infiltrates were more abundant in the kidney tissue of the control group than in that of the good-prognosis group (**Figures 4A, B**). When patients were stratified by median values, the groups with increased intrarenal infiltration of CD45^+^, CD20^+^, and CD3^+^ cells showed a significantly higher risk of progression to ESKD and composite renal outcomes, as demonstrated using Kaplan-Meier curves (**Figure 5**). After adjusting for clinical variables and histologic grade, the lower proportions of CD45^+^ (aOR, 0.77; 95% CI, 0.60–0.98; *P* = 0.03) and CD3^+^ (aOR, 0.84; 95% CI, 0.71–0.98; *P* = 0.03) cells were independently associated with good prognosis (**Table 4**). Ki-67^+^ cells were not significantly associated with prognosis in any of the subgroups (**Table 4**).

**Table 4:** Intrarenal leukocytes infiltration and Ki-67 positive cells as predictors of good prognosis.

Variables	Univariable		Multivariable	
	OR (95% CI)	p value	OR (95% CI)	p value
All patients				
CD45^+^ cells, %	0.77 (0.64 – 0.92)	0.004	0.77 (0.60 – 0.98)	0.033
CD20^+^ cells, %	0.71 (0.52 – 0.96)	0.027	0.90 (0.62 – 1.29)	0.563
CD3^+^ cells, %	0.80 (0.71 – 0.91)	<0.001	0.84 (0.71 – 0.98)	0.029
Ki-67^+^ cells, %	0.95 (0.47 – 1.95)	0.896	0.83 (0.35 – 1.95)	0.668
Patients receiving immunosuppressive therapy				
CD45^+^ cells, %	0.80 (0.65 – 1.00)	0.049	0.84 (0.62 – 1.14)	0.263
CD20^+^ cells, %	0.88 (0.63 – 1.23)	0.443	1.27 (0.84 – 1.94)	0.261
CD3^+^ cells, %	0.83 (0.70 – 0.98)	0.029	0.84 (0.66 – 1.07)	0.166
Ki-67^+^ cells, %	0.91 (0.43 – 1.90)	0.794	0.91 (0.33 – 2.55)	0.862
Patients receiving supportive care only				
CD45^+^ cells, %	0.40 (0.23 – 0.68)	<0.001	0.55 (0.29 – 1.03)	0.063
CD20^+^ cells, %	0.15 (0.05 – 0.41)	< 0.001	0.22 (0.07 – 0.73)	0.014
CD3^+^ cells, %	0.68 (0.55 – 0.86)	0.001	0.78 (0.58 – 1.04)	0.085
Ki-67^+^ cells, %	0.45 (0.02 – 10.62)	0.618	0.56 (0.01 – 37.50)	0.788

Adjusted for age, sex, BMI, MAP, hypertension, anemia, eGFR, uPCR, hematuria, and histologic grade (plus immunosuppressive therapy in the overall cohort).

Among patients receiving immunosuppressive therapy, CD45^+^ and CD3^+^ cell infiltrates were more abundant in the control group than in the good-prognosis group (**Figure 4C**). CD3^+^ cell infiltration showed a negative association with good prognosis in the univariable analysis but lost significance after adjustment (**Table 4**). Consequently, the extent of intrarenal leukocyte infiltration did not independently predict good prognosis in patients receiving immunosuppressive therapy.

Among those receiving supportive care only, CD45^+^, CD20^+^, and CD3^+^ cell infiltrates were significantly more abundant in the control group than in the good-prognosis group (**Figure 4D**). CD45^+^, CD20^+^, and CD3^+^ cell infiltrations were inversely associated with good prognosis in the univariable analysis. However, after adjustment, only CD20^+^ cell infiltration remained independently and negatively associated with good prognosis (aOR, 0.22; 95% CI, 0.07–0.73; *P* = 0.01), whereas CD45^+^ and CD3^+^ cells showed only borderline significance (**Table 4**).

### 3.6 Sensitivity analyses using hard renal outcomes and absolute proteinuria at 1 year

To further assess whether serum TGF-β1 and MCP-1 predict outcomes beyond baseline disease severity, we performed sensitivity analyses using hard renal outcomes and absolute uPCR at 1 year as alternative endpoints (**Supplementary Tables 9**, **10**). In Cox proportional hazards regression analyses for the composite renal outcome, higher serum TGF-β1 levels were independently associated with a lower risk in both the overall cohort (adjusted hazard ratio [aHR], 0.95; 95% CI, 0.91–0.99; P = 0.007) and the immunosuppressive therapy subgroup (aHR, 0.89; 95% CI, 0.82–0.95; P < 0.001) after adjusting for clinical variables and histologic grade, consistent with the primary analysis showing that higher serum TGF-β1 was associated with good prognosis (aOR, 1.16). Higher serum MCP-1 levels were independently associated with a higher risk of the composite renal outcome in both the overall cohort (aHR, 1.007; 95% CI, 1.002–1.013; P = 0.013) and the immunosuppressive therapy subgroup (aHR, 1.008; 95% CI, 1.001–1.016; P = 0.036). In contrast, neither biomarker was significantly associated with hard renal outcomes in the supportive care subgroup after adjustment. In linear regression analyses for absolute uPCR at 1 year, higher serum TGF-β1 (adjusted β, −0.037; 95% CI, −0.067 to −0.007; P = 0.016) and higher serum MCP-1 (adjusted β, 0.006; 95% CI, 0.001 to 0.011; P = 0.014) independently predicted lower and higher absolute uPCR at 1 year, respectively, among patients receiving immunosuppressive therapy, whereas neither biomarker showed significant association with uPCR in the supportive care subgroup.

## 4 Discussion

In this prospective cohort study of biopsy-proven IgAN, we identified serum cytokines and intrarenal leukocyte infiltration as potential biomarkers for predicting prognosis and response to immunosuppressive therapy. Among patients receiving immunosuppressive therapy, higher serum TGF-β1 and lower serum MCP-1 levels were independently associated with good prognosis after adjustment for clinical variables and pathologic grade, whereas intrarenal CD20^+^ cell infiltration was independently associated with prognosis in patients receiving supportive care only.

Although several biomarkers have been associated with prognosis, those predicting treatment response remain poorly defined (19–21). Recent studies suggest that urinary soluble CD163 suppression (22) and a lower neutrophil-to-lymphocyte ratio are related to better corticosteroid response (23). In contrast, the intensity of macrophage infiltration (24) and fibroblast-specific protein-1-positive cells (25) may predict the response to immunosuppressive therapy. However, the clinical use of these biomarkers remains limited.

Our findings suggest that systemic cytokine and chemokine profiles may serve as biomarkers for predicting favorable prognosis among patients receiving immunosuppressive therapy. Higher serum TGF-β1 and lower serum MCP-1 levels were independently associated with favorable outcomes in patients receiving immunosuppressive therapy. Serum TGF-β1 levels were higher in the good-prognosis group, whereas urine TGF-β1 levels showed the opposite pattern. TGF-β1 is a central mediator of renal fibrosis and a key driver of chronic kidney disease progression (26). Therefore, our findings may appear to be biologically counterintuitive. Although the underlying mechanism of these findings remains to be fully elucidated, we propose a possible explanation that reflects the dual roles of TGF-β1 (27). Most circulating TGF-β1 exists in a latent form and is converted to the active form at sites of tissue injury; total TGF-β1 measurement usually includes both forms through acid activation. Latent TGF-β1 exerts anti-inflammatory and antifibrotic effects by inducing Smad7-mediated inhibition of nuclear factor κB signaling and TGF-β1 activation (28, 29). Furthermore, active TGF-β1 signaling within the physiologic range promotes autophagy that degrades excess collagen (30). In contrast, locally excessive TGF-β1 activation promotes mesangial proliferation, extracellular matrix accumulation, and fibrosis (31), thereby contributing to disease progression. Accordingly, the serum level of TGF-β1 may reflect its protective effects through its latent form, whereas the urinary level may indicate local excess activation. However, several technical limitations should be acknowledged. The ELISA used in this study measured total TGF-β1 after acid activation and could not distinguish between latent and active isoforms. Moreover, serum TGF-β1 levels can be substantially influenced by platelet degranulation during blood clotting, as platelets are a major source of circulating TGF-β1. Variations in sample processing, including differences in clotting time or time to protocolized storage, may therefore affect measured concentrations. These factors warrant consideration in interpreting absolute serum TGF-β1 values, and further studies using plasma samples or isoform-specific assays are needed to clarify whether our findings reflect the effects of latent TGF-β1.

MCP-1 is a key chemokine secreted by renal tubular epithelial cells in response to inflammatory cytokines (32), promoting monocyte recruitment and C-C chemokine receptor type 2 (CCR2)-mediated signaling involved in renal fibrosis (33). Most studies investigating MCP-1 in IgAN have primarily focused on urine (20, 34, 35). A recent study showed that the MCP-1–CCR2 axis promotes migration of γδ1 T cells into the kidney, which correlates with severity of renal damage in IgAN (36). The authors noted increased serum MCP-1 levels, but no supporting data were provided. In our study, serum and urine MCP-1 levels were associated with good prognosis in patients receiving immunosuppressive therapy; however, the association for urine MCP-1 lost significance after adjustment, suggesting it mainly reflects tubulointerstitial injury or local inflammatory burden already captured by clinical and pathologic factors. The predictive ability of serum MCP-1 was modest, and its clinical utility requires further validation.

Intrarenal leukocyte infiltration was associated with poor prognosis overall but did not independently predict good prognosis among patients receiving immunosuppressive therapy beyond clinical variables and pathologic grade. Interstitial inflammation was excluded from the Oxford classification because of its strong correlation with interstitial fibrosis (37); however, some studies reported that it remains independently associated with IgAN progression (38, 39). In our cohort, patients receiving immunosuppressive therapy presented with significantly lower baseline eGFR and higher baseline proteinuria. Our finding that leukocyte infiltration lacked independent predictive value in the immunosuppressive subgroup seems to suggest that in advanced stages with established fibrosis, intrarenal leukocyte accumulation may reflect chronic inflammation associated with structural damage, rather than active, treatment-responsive inflammation. Because the Haas classification does not account for interstitial fibrosis, further studies are needed to elucidate the prognostic significance of leukocyte infiltration in relation to the histologic grade or fibrosis severity in IgAN.

CD20^+^ B cells were closely associated with poor prognosis in patients who received supportive care only. The pathogenesis of IgAN has traditionally been explained by the pathway of mesangial IgA immune complex deposition leading to mesangial cell activation and subsequent cytokine release and fibrosis (40). Consequently, most studies have focused on glomerular inflammation and the infiltration of interstitial macrophages and T-cell subpopulations. In contrast, B cells have mainly been investigated from the perspective of systemic mucosal immune dysregulation. However, CD20^+^ B cells constitute a component of the interstitial inflammatory infiltrates (41). Recent studies reported that peripheral follicular helper T-cell and intrarenal B-cell infiltration are correlated with renal dysfunction in IgAN (42), and that interstitial B-cell infiltration is associated with fibrosis through the production of profibrotic cytokines and chemokines (41). Further studies are needed to clarify the role of intrarenal infiltrated B cells in the pathogenesis and prognosis of IgAN.

Our study has certain limitations. First, as this was an observational study, treatment allocation was not randomized, and substantial differences in baseline characteristics between the good-prognosis and control groups might have introduced residual confounding. Although the major associations were statistically significant after adjustment, definite causality could not be established. Furthermore, the associations identified in the immunosuppressive therapy subgroup reflect prognostic value within treated patients rather than a confirmed treatment-response interaction. Accordingly, we use the term “good prognosis” rather than “treatment response” throughout this manuscript. Additionally, the immunosuppressive regimens used in this study were heterogeneous, encompassing several distinct monotherapies and combination protocols that may have different immunologic mechanisms and therapeutic effects. Because treatment strategies were individualized according to disease severity and prevailing clinical practice during the study period, subgroup analyses according to specific immunosuppressive regimens were limited by sample size. Second, this study was conducted at a single center, which may limit the generalizability of the findings, particularly given the potential differences in clinical practice patterns and disease characteristics across races and countries. Third, we used the criteria of good prognosis 1 year after kidney biopsy, defined by proteinuria reduction and preserved eGFR, as a short-term surrogate outcome. Although less definitive, this approach was chosen because IgAN usually progresses slowly, hard outcomes are infrequent, and short-term changes in proteinuria and eGFR are validated predictors of long-term prognosis (6). Moreover, our study focused on identifying biomarkers of favorable prognosis within each treatment group, for which early outcomes are more appropriate. Fourth, we used the Haas classification because the Oxford MEST-C classification had not yet been fully implemented at our institution during the study enrollment period (2010–2020). Although a previous study demonstrated comparable prognostic performance between the two classifications (43), the Haas classification does not separately evaluate active and chronic lesions. Exploratory analysis separating Haas grades IV (diffuse active) and V (advanced chronic) suggested that grade IV was associated with a higher rate of good prognosis than grade V, and serum TGF-β1 showed a consistent association with good prognosis across both grades, although these findings were limited by small subgroup sizes. In our study, a high Haas histological grade (grades IV and V) was a robust independent predictor of poor prognosis. However, future prospective validation using the full MEST-C scoring components is warranted to better differentiate active immunologic lesions from chronic fibrotic processes. Fifth, the events per variable in the immunosuppressive therapy subgroup analyses were below the conventional threshold, raising the possibility of model instability. To mitigate this concern, we assessed multicollinearity, constructed parsimonious models with reduced covariates, and performed bootstrap internal validation. Serum TGF-β1 remained significant across all parsimonious models, and the optimism-corrected AUROC confirmed that the incremental predictive value of the biomarkers was maintained after accounting for overfitting. Although the improvement in AUROC did not reach statistical significance, continuous NRI and IDI demonstrated significant reclassification improvement, suggesting clinically meaningful incremental value despite the limited sample size. Furthermore, sensitivity analyses using Cox regression for hard renal outcomes and linear regression for absolute uPCR at 1 year yielded results consistent with the primary analysis, supporting the robustness of our findings. Nevertheless, external validation in an independent cohort is warranted to confirm the generalizability of these results.

## 5 Conclusions

We identified serum TGF-β1 and MCP-1 as promising biomarkers for predicting favorable prognosis among patients with IgAN receiving immunosuppressive therapy. In addition, intrarenal leukocyte infiltration, including CD20^+^ cells, may serve as a biomarker for predicting prognosis of IgAN. These findings support the potential value of integrating immunological profiling into risk stratification and may help inform individualized treatment decisions. Further multicenter studies with larger cohorts are warranted to validate these results and explore the mechanistic basis of cytokine- and immune cell-driven disease progression.

## Data Availability

The raw data supporting the conclusions of this article will be made available by the authors, without undue reservation.
