# Supplementary material for: Biomarkers predicting good prognosis among patients receiving immunosuppressive treatment in IgA nephropathy: the promising role of serum TGF-β1 and MCP-1
Source: Front Immunol. 2026 Jun 24;17:1852831. doi: 10.3389/fimmu.2026.1852831 (PMC13341302; doi:10.3389/fimmu.2026.1852831)
Supplement: Supplementary file 1 [file DataSheet1.pdf]

Supplemental Materials

Supplement to: Biomarkers predicting response to immunosuppressive treatment in IgA nephropathy: The promising role of serum TGF-β1 and MCP-1

Junseok Jeon et al.

Table of Contents

Supplementary Table S1. Variance inflation factors for covariates in multivariable logistic regression models

Supplementary Table S2. Baseline characteristics of patients receiving immunosuppressive therapy (n = 96)

Supplementary Table S3. Baseline characteristics of patients receiving supportive care only (n = 106)

Supplementary Table S4. Comparison of outcomes and biomarker levels between Haas grade IV and grade V, stratified by treatment group and prognosis

Supplementary Table S5. Parsimonious logistic regression models for good prognosis among patients receiving immunosuppressive therapy

Supplementary Table S6. Bootstrap internal validation of logistic regression models for good prognosis among patients receiving immunosuppressive therapy

Supplementary Table S7. Comparison of predictive performance of logistic regression models for good prognosis among patients receiving immunosuppressive therapy

Supplementary Table S8. Net reclassification improvement and integrated discrimination improvement for predicting good prognosis among patients receiving immunosuppressive therapy

Supplementary Table S9. Cox proportional hazards regression analysis of serum TGF-β1 and MCP-1 for predicting hard renal outcomes

Supplementary Table S10. Linear regression analysis of serum TGF-β1 and MCP-1 for predicting absolute urine protein-to-creatinine ratio at 1 year after kidney biopsy

Supplementary Figure S1. Serum and urine cytokine/chemokine levels in all patients

Supplementary Figure S2. Kidney outcomes according to serum cytokine levels in patients receiving immunosuppressive therapy

Supplementary Table S1. Variance inflation factors for covariates in multivariable logistic regression models

Variable	All patients	IS subgroup
Age	1.56	1.59
Male sex	1.22	1.23
BMI	1.22	1.38
MAP	1.78	1.87
Hypertension	1.42	1.30
Anemia	1.44	1.72
eGFR	1.85	1.79
uPCR	1.38	1.49
Minimal hematuria	1.19	1.20
Histologic grade	1.29	1.19
IS therapy	1.36	—
Serum TGF-β1	1.39	1.39
Serum MCP-1	1.13	1.18

All VIF values were below 2.0, indicating no substantial multicollinearity among the covariates. VIF, variance inflation factor; BMI, body mass index; eGFR, estimated glomerular filtration rate; IS, immunosuppressive; MAP, mean arterial pressure; MCP-1, monocyte chemoattractant protein-1; TGF-β1, transforming growth factor-β1; uPCR, urine protein-to-creatinine ratio.

Supplementary Table S2. Baseline characteristics of patients receiving immunosuppressive therapy (n = 96)

	**Control (n = 38)**	**Good prognosis (n = 58)**	***P* value**
Age (years)	43.2 ± 12.9	43.4 ± 11.9	0.944
Male sex	20 (52.6)	32 (55.2)	0.807
BMI (kg/m^2)	22.5 ± 3.6	23.2 ± 3.3	0.358
HTN	22 (73.7)	19 (32.8)	0.015
MAP (mmHg)	96.8 ± 12.6	91.3 ± 11.8	0.037
Anemia[a]	28 (73.7)	32 (55.2)	0.067
eGFR (mL/min/1.73 m^2)	48.3 ± 24.7	61.7 ± 28.7	0.017
uPCR (mg/mg)	2.8 ± 2.1	2.0 ± 1.7	0.085
Negative or minimal hematuria[b]	6 (15.8)	12 (20.7)	0.547
High histological grade[c]	30 (78.9)	31 (53.4)	0.011

Values are reported as mean ± standard deviation for continuous variables and as number (percentage) for categorical variables.

[a]Anemia was defined as hemoglobin level <13.5 mg/dL in men and <12 mg/dL in women.

[b]Negative or minimal hematuria: 0–11 red blood cells per high-power field.

[c]Haas grade IV or V.

BMI, body mass index; HTN, hypertension; MAP, mean arterial pressure; eGFR, estimated glomerular filtration rate; uPCR, urine protein-to-creatinine ratio

Supplementary Table S3. Baseline characteristics of patients receiving supportive care only (n = 106)

	**Control (n = 62)**	**Good prognosis (n = 44)**	***P* value**
Age (years)	45.0 ± 12.7	40.1 ± 15.0	0.084
Male sex	28 (45.2)	20 (45.5)	0.976
BMI (kg/m^2)	23.9 ± 3.4	23.7 ± 4.7	0.817
HTN	36 (58.1)	15 (34.1)	0.015
MAP (mmHg)	93.1 ± 9.5	88.0 ± 9.1	0.007
Anemia[a]	31 (50.0)	18 (40.9)	0.355
eGFR (mL/min/1.73 m^2)	62.2 ± 30.9	77.6 ± 31.2	0.014
uPCR (mg/mg)	1.6 ± 1.1	1.0 ± 1.0	0.002
Negative or minimal hematuria[b]	25 (40.3)	10 (22.7)	0.058
High histological grade[c]	29 (46.8)	10 (22.7)	0.011

Values are reported as mean ± standard deviation for continuous variables and as number (percentage) for categorical variables.

[a]Anemia was defined as hemoglobin level <13.5 mg/dL in men and <12 mg/dL in women.

[b]Negative or minimal hematuria: 0–11 red blood cells per high-power field.

[c]Haas grade IV or V.

BMI, body mass index; HTN, hypertension; MAP, mean arterial pressure; eGFR, estimated glomerular filtration rate; uPCR, urine protein-to-creatinine ratio

Supplementary Table S4. Comparison of outcomes and biomarker levels between Haas grade IV and grade V, stratified by treatment group and prognosis

	**Immunosuppressive therapy**		**Supportive care only**	
	**Haas IV (n = 36)**	**Haas V (n = 25)**	**Haas IV (n = 24)**	**Haas V (n = 15)**
Good prognosis, n (%)	21 (58.3)	10 (40.0)	8 (33.3)	2 (13.3)
ESKD events, n (%)	7 (19.4)	13 (52.0)	3 (12.5)	5 (33.3)
**Serum TGF-β1, ng/mL**				
Control	21.0 ± 3.6	17.9 ± 6.9	21.0 ± 10.0	22.8 ± 9.5
Good prognosis	23.4 ± 6.8	26.4 ± 9.2	29.5 ± 10.4	21.3 ± 9.5
**Serum MCP-1, pg/mL**				
Control	76.3 ± 50.0	103.9 ± 40.1	90.2 ± 48.5	84.9 ± 58.2
Good prognosis	81.3 ± 37.2	81.6 ± 50.4	93.8 ± 55.2	75.7 ± 55.0

Values for continuous variables are presented as mean ± standard deviation and categorical variables as number (percentage). Haas grade IV reflects predominantly active proliferative lesions and grade V reflects predominantly chronic sclerotic lesions. Serum TGF-β1 and MCP-1 data were available for a subset of patients (see Methods). ESKD, end-stage kidney disease; MCP-1, monocyte chemoattractant protein-1; TGF-β1, transforming growth factor-β1.

Supplementary Table S5. Parsimonious logistic regression models for good prognosis among patients receiving immunosuppressive therapy

Biomarker	Model	n	EPV	Adjusted OR (95% CI)	P
Serum TGF-β1	Full (10 covariates)	86	2.9	1.16 (1.04–1.31)	0.010
	Parsimonious A (4 covariates)	86	6.4	1.11 (1.02–1.21)	0.011
	Parsimonious B (3 covariates)	86	8.0	1.11 (1.03–1.20)	0.009
Serum MCP-1	Full (10 covariates)	89	3.0	0.99 (0.97–1.00)	0.016
	Parsimonious A (4 covariates)	89	6.6	0.99 (0.98–1.00)	0.058
	Parsimonious B (3 covariates)	89	8.2	0.99 (0.98–1.00)	0.045

Full model includes age, sex, BMI, MAP, hypertension, anemia, eGFR, uPCR, hematuria, and histologic grade. Parsimonious A includes hypertension, eGFR, uPCR, and histologic grade (variables with $P < 0.1$ in univariable analysis). Parsimonious B includes eGFR, uPCR, and histologic grade. CI, confidence interval; EPV, events per variable; IS, immunosuppressive; MCP-1, monocyte chemoattractant protein-1; OR, odds ratio; TGF-β1, transforming growth factor-β1.

Supplementary Table S6. Bootstrap internal validation of logistic regression models for good prognosis among patients receiving immunosuppressive therapy

Model	Apparent AUROC	Optimism	Corrected AUROC
Clinical + pathologic only	0.8194	0.0827	0.7367
+ Serum TGF-β1	0.8328	0.0955	0.7372
+ Serum MCP-1	0.8209	0.0950	0.7259
+ Serum TGF-β1 + Serum MCP-1	0.8501	0.0993	0.7507

Internal validation was performed using 1,000 bootstrap resamples. Optimism was calculated as the difference between the bootstrap training AUROC and the AUROC obtained by applying the bootstrap model to the original dataset. The optimism-corrected AUROC was calculated by subtracting the mean optimism from the apparent AUROC. AUROC, area under the receiver operating characteristic curve; MCP-1, monocyte chemoattractant protein-1; TGF-β1, transforming growth factor-β1.

Supplementary Table S7. Comparison of predictive performance of logistic regression models for good prognosis among patients receiving immunosuppressive therapy

Model	AUROC (95% CI)	ΔAUROC	P value	Reference
Model 1: Clinical + pathologic	0.789 (0.682–0.884)	—	—	—
Model 2: + Serum TGF-β1	0.831 (0.736–0.919)	0.042	0.180	Model 1
Model 3: + TGF-β1 + MCP-1	0.850 (0.755–0.929)	0.061	0.062	Model 1
Model 3 vs Model 2		0.019	0.371	Model 2

95% CI for AUROC was calculated using 1,000 bootstrap resamples. P values for ΔAUROC were calculated using the bootstrap method. Model 1 includes age, sex, BMI, MAP, hypertension, anemia, eGFR, uPCR, hematuria, and histologic grade. Model 2 adds serum TGF-β1 to Model 1. Model 3 adds serum TGF-β1 and serum MCP-1 to Model 1. AUROC, area under the receiver operating characteristic curve; CI, confidence interval; MCP-1, monocyte chemoattractant protein-1; TGF-β1, transforming growth factor-β1.

Supplementary Table S8. Net reclassification improvement and integrated discrimination improvement for predicting good prognosis among patients receiving immunosuppressive therapy

		Continuous NRI		IDI	
**Comparison**	**Reference**	**Estimate (95% CI)**	**P**	**Estimate (95% CI)**	**P**
Model 2	Model 1	0.651 (0.251–1.055)	0.003	0.083 (0.028–0.145)	0.006
Model 3	Model 1	0.661 (0.229–1.070)	0.003	0.123 (0.056–0.199)	<0.001

Model 1: clinical and pathologic variables only. Model 2: Model 1 + serum TGF-β1. Model 3: Model 1 + serum TGF-β1 + serum MCP-1. Continuous NRI and IDI were calculated with 95% CIs derived from 1,000 bootstrap resamples. CI, confidence interval; IDI, integrated discrimination improvement; MCP-1, monocyte chemoattractant protein-1; NRI, net reclassification improvement; TGF-β1, transforming growth factor-β1.

Supplementary Table S9. Cox proportional hazards regression analysis of serum TGF-β1 and MCP-1 for predicting hard renal outcomes

				Unadjusted		Adjusted	
**Subgroup**	**Variable**	**n**	**Events**	**HR (95% CI)**	**P**	**HR (95% CI)**	**P**
**ESRD**							
All patients	Serum TGF-β1	187	29	0.93 (0.88–0.97)	0.003	0.97 (0.92–1.02)	0.194
	Serum MCP-1	187	31	1.007 (1.000–1.014)	0.060	1.008 (1.000–1.016)	0.052
IS subgroup	Serum TGF-β1	86	18	0.94 (0.89–1.00)	0.054	0.98 (0.90–1.06)	0.551
	Serum MCP-1	89	20	1.010 (1.001–1.019)	0.038	1.010 (1.000–1.020)	0.051
SC subgroup	Serum TGF-β1	101	11	0.91 (0.84–0.99)	0.030	0.97 (0.90–1.05)	0.478
	Serum MCP-1	98	11	1.001 (0.989–1.013)	0.873	0.996 (0.980–1.013)	0.655
**Composite renal outcome**							
All patients	Serum TGF-β1	187	59	0.93 (0.90–0.97)	<0.001	0.95 (0.91–0.99)	0.007
	Serum MCP-1	187	61	1.007 (1.002–1.012)	0.005	1.007 (1.002–1.013)	0.013
IS subgroup	Serum TGF-β1	86	32	0.91 (0.87–0.96)	<0.001	0.89 (0.82–0.95)	<0.001
	Serum MCP-1	89	34	1.007 (1.000–1.015)	0.058	1.008 (1.001–1.016)	0.036
SC subgroup	Serum TGF-β1	101	27	0.96 (0.91–1.01)	0.091	0.99 (0.94–1.04)	0.647
	Serum MCP-1	98	27	1.008 (1.000–1.015)	0.039	1.004 (0.994–1.014)	0.455

Adjusted for age, sex, BMI, MAP, hypertension, anemia, eGFR, uPCR, hematuria, and histologic grade (plus immunosuppressive therapy in the overall cohort). Composite renal outcome is defined as ESRD or ≥50% decline in eGFR from baseline. CI, confidence interval; ESRD, end-stage kidney disease; HR, hazard ratio; IS, immunosuppressive; MCP-1, monocyte chemoattractant protein-1; SC, supportive care; TGF-β1, transforming growth factor-β1.

Supplementary Table S10. Linear regression analysis of serum TGF-β1 and MCP-1 for predicting absolute urine protein-to-creatinine ratio at 1 year after kidney biopsy

			Unadjusted		Adjusted	
**Subgroup**	**Variable**	**n**	**β (95% CI)**	**P**	**β (95% CI)**	**P**
All patients	Serum TGF-β1	187	−0.027 (−0.045 to −0.008)	0.006	−0.015 (−0.031 to 0.002)	0.076
	Serum MCP-1	187	0.003 (0.000 to 0.007)	0.057	0.003 (0.000 to 0.006)	0.034
IS subgroup	Serum TGF-β1	86	−0.020 (−0.054 to 0.014)	0.252	−0.037 (−0.067 to −0.007)	0.016
	Serum MCP-1	89	0.008 (0.002 to 0.014)	0.014	0.006 (0.001 to 0.011)	0.014
SC subgroup	Serum TGF-β1	101	−0.031 (−0.052 to −0.010)	0.004	−0.004 (−0.022 to 0.013)	0.618
	Serum MCP-1	98	0.000 (−0.004 to 0.004)	0.953	0.001 (−0.002 to 0.003)	0.698

Adjusted for age, sex, BMI, MAP, hypertension, anemia, eGFR, uPCR, hematuria, and histologic grade (plus immunosuppressive therapy in the overall cohort). Composite renal outcome is defined as ESRD or ≥50% decline in eGFR from baseline. CI, confidence interval; ESRD, end-stage kidney disease; HR, hazard ratio; IS, immunosuppressive; MCP-1, monocyte chemoattractant protein-1; SC, supportive care; TGF-β1, transforming growth factor-β1.

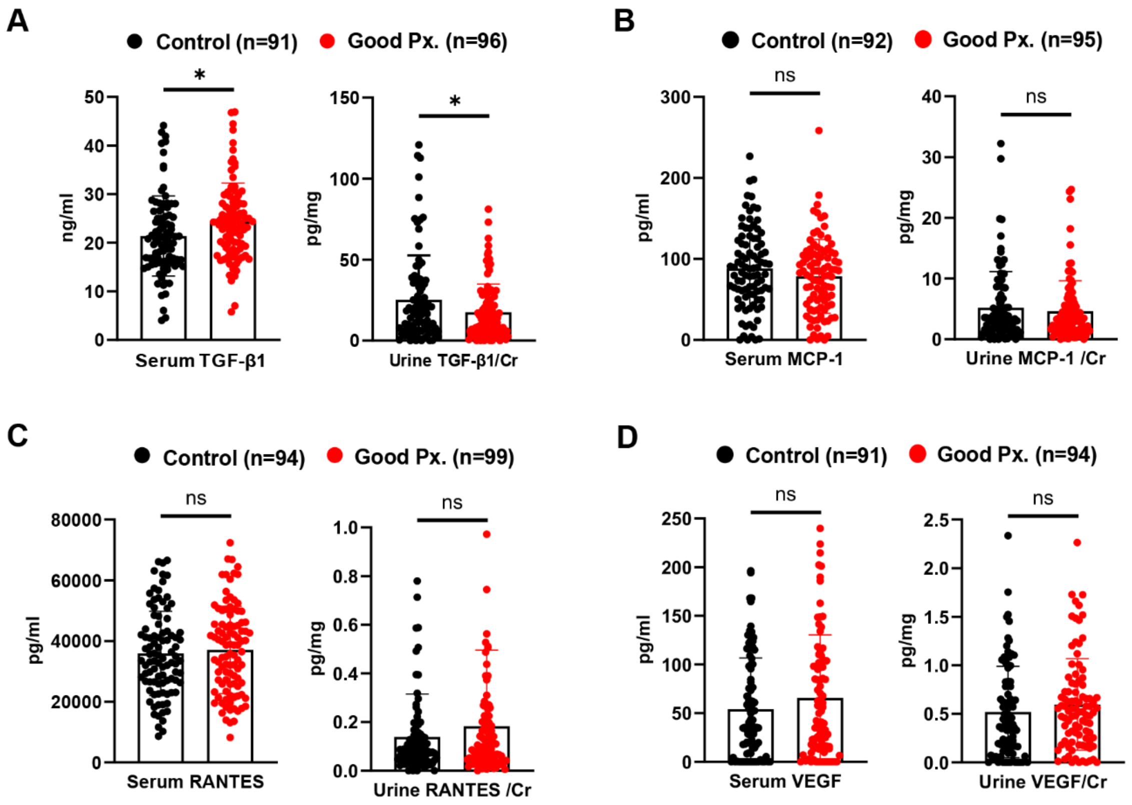

Supplementary Fig. S1. Serum and urine cytokine/chemokine levels in all patients.

Comparison of serum and urine cytokine/chemokine levels between the good-prognosis (blue) and control (black) groups among patients treated with immunosuppressive therapy. Panels show concentrations of (A) TGF-β1, (B) MCP-1, (C) RANTES, and (D) VEGF. Urine levels were normalized to the urine Cr concentration. Data are reported as individual patient values overlaid on bar plots representing the mean ± standard error of the mean. Statistical comparisons were performed using Student's t-test. For all graphs, NS, not significant; **P* < 0.05. TGF-β1, transforming growth factor-β1; Cr, creatinine; MCP-1, monocyte chemoattractant protein-1; Px, prognosis; RANTES, regulated on activation, normal T cell expressed and secreted; VEGF, vascular endothelial growth factor

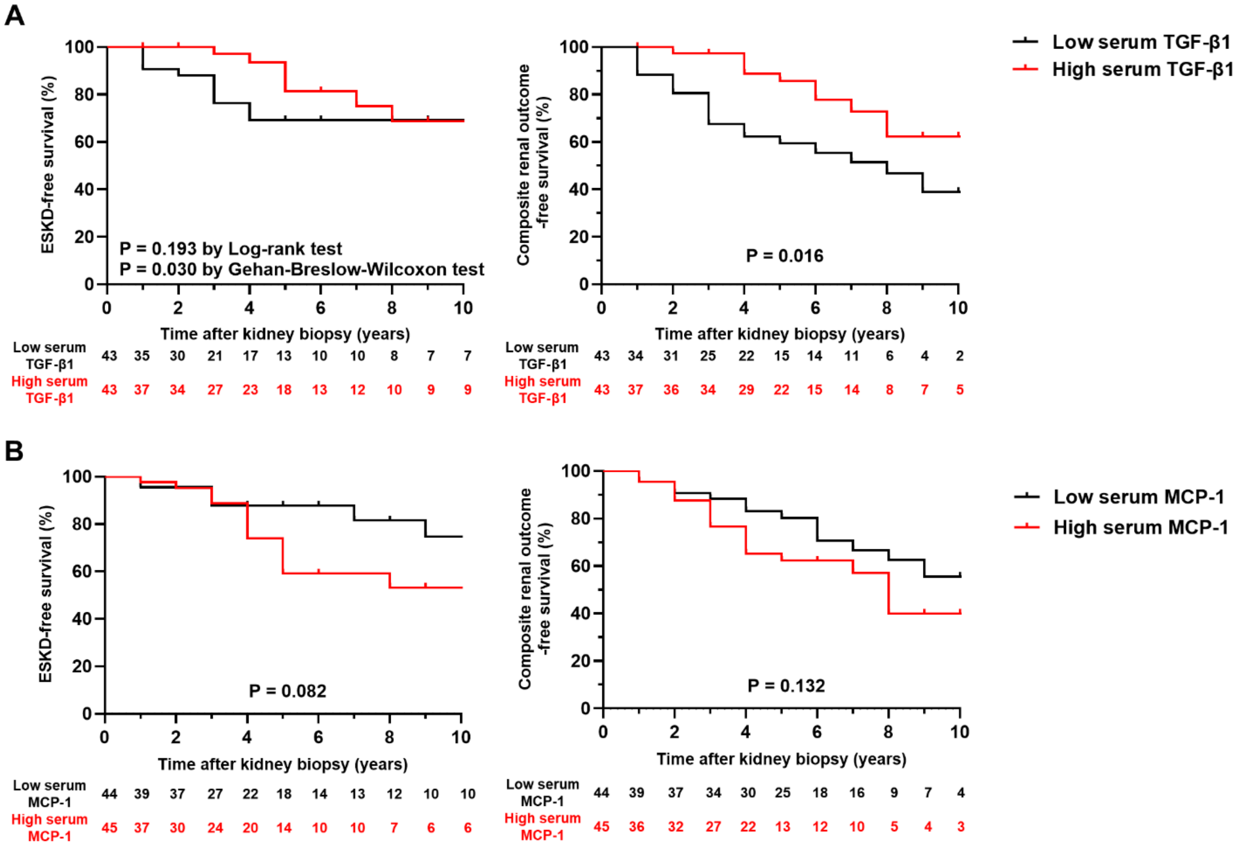

Supplementary Fig. S2. Kidney outcomes according to serum cytokine levels in patients receiving immunosuppressive therapy.

Kaplan-Meier survival curves for kidney outcomes in patients treated with immunosuppressive therapy, stratified by median serum cytokine levels. (A) ESKD-free survival (left) and composite renal outcome-free survival (right) according to serum TGF-β1 levels (low, n = 43; high, n = 43). (B) ESKD-free survival (left) and composite renal outcome-free survival (right) according to MCP-1 levels (low, n = 44; high, n = 45). *P* values were calculated using the log-rank test, except for ESKD-free survival in panel A, for which the Gehan-Breslow-Wilcoxon test was used because it gives greater weight to early events. ESKD, end-stage kidney disease; TGF-β1, transforming growth factor-β1; MCP-1, monocyte chemoattractant protein-1
